# Association between the type of physical activity and metabolic syndrome in middle-aged and older adult residents of a semi-mountainous area in Japan

**DOI:** 10.1186/s12199-021-00949-x

**Published:** 2021-04-10

**Authors:** Noriko Kudo, Ritsuko Nishide, Mayumi Mizutani, Shota Ogawa, Susumu Tanimura

**Affiliations:** Department of Public Health Nursing, Mie University Graduate School of Medicine, 2-174 Edobashi, Tsu, Mie 514-8507 Japan

**Keywords:** Exercise, Daily physical activity, Metabolic syndrome, Women

## Abstract

**Background:**

Physical activity is reported to prevent metabolic syndrome. However, it is unclear whether exercise or daily physical activity is more beneficial for residents of semi-mountainous areas. This study aimed to identify whether daily physical activity is more beneficial than exercise for the prevention of metabolic syndrome among middle-aged and older residents in semi-mountainous areas.

**Methods:**

We analyzed secondary data of 636 people who underwent a specific health checkup in a semi-mountainous area of Japan. Physical activity was classified into four types: inactivity (I-type; without exercise and without daily physical activity), only exercise (E-type; with exercise and without daily physical activity), only daily physical activity (D-type; without exercise and with daily physical activity), and full physical activity type (F-type; with exercise and with daily physical activity). We compared the means of risk factors for metabolic syndrome by these four types, followed by logistic regression analysis, to identify whether and to what extent the D-type was less likely to have metabolic syndrome than the E-type.

**Results:**

The prevalence of metabolic syndrome was 28.5% (men 45.7%, women 15.8%). The proportions of men with exercise and daily physical activity were 38.7% and 52.8%, respectively. For women, the proportions were 33.0% and 47.1%, respectively. In women, the D-type had the significantly lowest BMI, smallest waist circumference, highest HDL-C, and lowest prevalence of metabolic syndrome of the four types; the same was not observed in men. Additionally, D-type activity was more strongly associated with a reduced risk of metabolic syndrome than E-type activity in women (adjusted odds ratio 0.24; 95% confidence interval 0.06–0.85, *P* = 0.028).

**Conclusions:**

Compared to middle-aged and older women residents with exercise in a semi-mountainous area of Japan, those with daily physical activity may effectively prevent metabolic syndrome.

## Background

A recently published systematic review showed a positive association between physical inactivity and metabolic syndrome [1]. In 2016, the prevalence of insufficient physical activity was 35.5% among adults (age ≥ 18 years; 37.0% for women, 33.8% for men) in Japan, which is higher than the global average (27.5%) [2, 3]. Therefore, physical activity promotion is important for the prevention of metabolic syndrome and its components [4].

Physical activity refers to “any bodily movement produced by skeletal muscles requiring energy expenditure” [5, 6] and is classified into two types: daily physical activity and exercise [7]. Daily physical activity includes activities undertaken daily, such as working, performing household chores, and traveling, whereas exercise includes planned, structured, and repetitive activities, such as sports [5].

Systematic reviews and meta-analyses have shown that exercise [8, 9] and leisure-time physical activity [10] are beneficial in preventing and improving the major causes of death-related chronic illnesses, including metabolic syndrome. Besides the benefits of exercise, the health risks associated with inactivity [11] and the importance of interrupting sedentary behavior [12] have been added to the World Health Organization’s new guidelines and evidence profile on physical activity and sedentary behavior [13–15].

However, there is no robust evidence that examined which of the two types of activity, namely, exercise-related activity or daily physical activity, effectively prevents metabolic syndrome.

Several studies have shown that rural residents are less likely to be physically active than urban residents [16–18]. Rural areas tend to lack public transportation, such as buses and trains, and people in these regions typically depend on self-owned vehicles for transportation. Car ownership reduces opportunities for these individuals to engage in physical activity [19]. However, previous research has shown that hill slopes and beautiful landscapes encourage walking without using bicycles and that the possibility of “unconscious” physical training is protective against the exacerbation of diabetes [20]. Therefore, in semi-mountainous areas, physical activity that differs from that in flat terrains may have a potential preventive effect against metabolic syndrome. Thus, it is necessary to clarify the association between physical activity and metabolic syndrome in semi-mountainous areas.

This study aimed to identify whether daily physical activity without exercise has a greater association with the prevention of metabolic syndrome than exercise without daily physical activity for the middle-aged and older residents in a semi-mountainous area of Japan.

## Methods

### Participants and data sources

This cross-sectional study used data from a specific health checkup from July to November 2017, provided by the health department of a local authority at a semi-mountainous, agricultural, rural community in Japan. Based on the explanation by the Ministry of Agriculture, Forestry and Fisheries of Japan [21] and the Japanese Ministry of Health, Labour and Welfare (MHLW) [22], we defined a semi-mountainous area as a place mostly covered with slopes that was neither urban nor open plain. It is usually located on the upstream river and occupies about 70% of Japan’s territory.

The specific health checkup targeted residents aged 40–74 years covered by the national health insurance of Japan and were aimed at early detection and intervention for individuals with a high risk of metabolic syndrome [23]. The specific health checkup data included general information (e.g., age and sex), anthropometric measurements, laboratory values, and responses to a self-reported questionnaire on lifestyle behaviors [23]. Health check and questionnaire data were collected on the same day. Exclusion criteria were set as missing valuables, such as sex, age, or questions concerning physical activity.

### Explanatory variables: physical activity types

We used two dichotomized questions regarding physical activity [23] that were based on the physical activity standards for health specified by the MHLW in 2013 [7]. The questions assessed exercise and daily physical activity. The exercise was assessed using the following question (Q1): “Have you been exercising for at least 1 year, at least 2 days per week, or at least 30 min each time at an intensity that causes slight sweating?” with a dichotomized response of “yes” (with exercise) and “no” (without exercise). Daily physical activity was assessed using the following question (Q2): “Do you walk for at least 1 h every day or have equivalent physical activities in your daily life?” with a dichotomized response of “yes” (with daily physical activity) and “no” (without daily physical activity) [23].

We classified participants into the following four types according to their answers to the two dichotomized questions of exercise (Q1) and daily physical activity (Q2) (Table 1): inactivity type (I-type, without exercise and daily physical activity), only exercise (E-type, with exercise and without daily physical activity), only daily physical activity type (D-type, without exercise and with daily physical activity), and full physical activity (F-type, with exercise and daily physical activity).

**Table 1:** Category of physical activity type

		Q1. Exercise^a^	
		With	Without
**Q2. Daily physical activity**^**b**^	**With**	F-type	D-type
	**Without**	E-type	I-type

^a^“Q1. Exercise” denotes answers to “Have you been exercising for at least 1 year, at least 2 days per week, or at least 30 min each time at an intensity that causes slight sweating?”

^b^“Q2. Daily physical activity” denotes answers to “Do you walk for at least 1 h every day or have equivalent physical activities in your daily life?”                               “With” and “Without” denote affirmative and negative answers, respectively. F, D, E, and I denote full physical activity, only daily physical activity, only exercise, and inactivity, respectively

### Objective variables: metabolic syndrome and its risk factors

We used anthropometric measurements and laboratory values to assess metabolic syndrome and its risk factors. These variables included body mass index (BMI), waist circumference, systolic blood pressure, diastolic blood pressure, triglycerides, high-density lipoprotein cholesterol (HDL-C), low-density lipoprotein cholesterol (LDL-C), and glycated hemoglobin (HbA1c).

Participants were classified as having metabolic syndrome if they had an excessive waist circumference (≥ 85 cm for men, ≥ 90 cm for women) and met two or more of the following criteria. If only one criterion was met, it was classified as a pre-metabolic syndrome: (1) elevated blood pressure (≥ 130 mmHg, systolic; ≥ 85 mmHg, diastolic; or use of antihypertensive drugs); (2) reduced HDL-C levels (< 40 mg/dL), elevated triglyceride levels (≥ 150 mg/dL), or use of anti-hyperlipidemic drugs; and (3) elevated glucose levels (fasting blood glucose ≥ 110 mg/dL, HbA1c ≥ 6.1%, or use of antidiabetic drugs) [24]. According to the criteria of the Japanese MHLW, participants were assigned to four groups: metabolic syndrome, pre-metabolic syndrome, no metabolic syndrome, and undeterminable [24]. The MHLW considers metabolic and pre-metabolic syndromes as important targets to reduce the risk of metabolic syndrome-related diseases [25]. Therefore, in this study, we re-categorized the groups based on a dichotomized variable: “metabolic syndrome: case” (metabolic syndrome and pre-metabolic syndrome) and “metabolic syndrome: non-case” (non-metabolic syndrome). The participants in the undeterminable group were excluded from our analysis.

### Statistical analysis

Cases with missing values were excluded list-wise. The following analyses were stratified according to sex. First, we calculated the descriptive statistics for all variables. Then, we performed bivariate sex-stratified analyses using Fisher’s exact test or *t*-test to compare sociodemographic characteristics, metabolic syndrome status, and risk factors for metabolic syndrome with the participants’ physical activity (exercise and daily physical activity). Next, we performed one-way analyses of variance to demonstrate differences in the prevalence of metabolic syndrome and its risk factors among the four physical activity types by sex, assisted by the post hoc analyses of Tukey’s honest significant difference test. Lastly, we conducted binomial logistic regression analyses to identify whether and to what extent the D-type group was more or less likely to have metabolic syndrome than the E-type group. We calculated the unadjusted odds ratio (OR), adjusted OR (AOR), and 95% confidence interval (CI) for each variable. *P*-values of less than 0.05 were considered to indicate statistical significance. Data were analyzed using R version 3.6.1 [26].

## Results

### Participant characteristics and physical activities

The analysis dataset included data from 636 adults (269 men and 367 women) in the age group of 40–74 years [mean (standard deviation, SD) 66.1 (7.8) years for men, 66.3 (7.1) years for women; Table 2]. No data was excluded from the criteria. The prevalence of metabolic syndrome in men and women was 45.7% and 15.8%, respectively. The proportion of participants with exercise was 38.7% and 33.0% among men and women, respectively, and 52.8% and 47.1% of the male and female participants were reported with daily physical activity, respectively.

**Table 2:** Participant characteristics and physical activities (*n* = 636)

		Men (***n*** = 269, 42.3%)		Women (***n*** = 367, 57.7%)		***P*** for sex-related differences^**c**^
		***n***	(%)	***n***	(%)	
**Q1. Exercise**^**a**^						
With		104	(38.7)	121	(33.0)	0.154
without		165	(61.3)	246	(67.0)	
**Q2. Daily physical activity**^**a**^						
With		142	(52.8)	173	(47.1)	0.173
Without		147	(54.6)	194	(52.9)	
**Metabolic syndrome**^**b**^						
Case		123	(45.7)	58	(15.8)	**<0.001**
Non-case		146	(54.3)	309	(84.2)	
		**mean**	**(SD)**	**mean**	**(SD)**	
Age, years		66.1	(7.8)	66.3	(7.1)	**0.001**
Body mass index, kg/m^2^		23.3	(3.0)	22.1	(3.5)	**0.017**
Waist circumference, cm		85.0	(8.0)	81.4	(9.8)	**0.014**
Systolic blood pressure, mmHg		131.3	(16.5)	129.5	(16.0)	**0.004**
Diastolic blood pressure, mmHg		76.5	(11.1)	73.3	(10.5)	**0.014**
Triglyceride, mg/dL		135.5	(88.2)	102.4	(57.2)	0.088
HDL-C, mg/dL		58.2	(15.8)	68.7	(16.4)	0.052
LDL-C, mg/dL		117.6	(30.7)	119.4	(29.7)	**0.005**
HbA1c, %		6.0	(0.8)	5.8	(0.5)	**0.011**

*HDL-C* high-density lipoprotein cholesterol, *LDL-C* low-density lipoprotein cholesterol, *HbA1c* glycated hemoglobin

^a^”Q1. Exercise" and "Q2. Daily physical activity”: see Table 1

^b^Metabolic syndrome: case (metabolic syndrome and pre-metabolic syndrome), non-case (non-metabolic syndrome)

^c^*P* for sex-related difference, independent *t*-test for continuous variables, or Fisher's exact test for categorical variables. Boldface indicates statistically significant differences

### Proportion of participants with metabolic syndrome by sex and physical activity

The proportion of participants with metabolic syndrome differed according to physical activity (with/without) and type (Table 3). A lower proportion (9.2%) of women with daily physical activity had metabolic syndrome than women without daily physical activity (21.6%). Similarly, men with daily physical activity had a lower prevalence of metabolic syndrome (38.7%) than those without daily physical activity (53.5%). The prevalence of metabolic syndrome among women according to the physical activity type significantly differed between the D-type and I-type groups (*P* < 0.001). Although the prevalence among men was significantly different among the four activity types (*P* = 0.033), the significance did not persist in multiple comparisons (Table 3).

**Table 3:** Proportions of metabolic syndrome in analyses stratified by sex and physical activity

				Metabolic syndrome^**b**^				
			Total	Case		Non-case		***P***
			***n***	***n***	(%)	***n***	(%)	
**Men**			269	123	(45.7)	146	(54.3)	
**Q1. Exercise**^**a**^								
With			104	40	(38.5)	64	(61.5)	0.060
Without			165	83	(50.3)	82	(49.7)	
**Q2. Daily physical activity**^**a**^								
With			142	55	(38.7)	87	(61.3)	**0.020**
Without			127	68	(53.5)	59	(46.5)	
**Physical activity type**								
I-type^a^			112	63	(56.3)	49	(43.8)	**0.033**
E-type^a^			15	5	(33.3)	10	(66.7)	
D-type^a^			53	20	(37.7)	33	(62.3)	
F-type^a^			89	35	(39.3)	54	(60.7)	
**Women**			367	58	(15.8)	309	(84.2)	
**Q1. Exercise**^**a**^								
With			121	18	(14.9)	103	(85.1)	0.764
Without			246	40	(16.3)	206	(83.7)	
**Q2. Daily physical activity**^**a**^								
With			173	16	(9.2)	157	(90.8)	**0.001**
Without			194	42	(21.6)	152	(78.4)	
**Physical activity type**								
I-type^a^			162	35	(21.6)	127	(78.4)^d^	**0.005**
E-type^a^			32	7	(21.9)	25	(78.1)	
D-type^a^			84	5	(6.0)	79	(94.0)^d^	
F-type^a^			89	11	(12.4)	78	(87.6)	

^a^Q1. Exercise, and Q2. Daily physical activity, I-type, E-type, D-type, and F-type: see Table 1

^b^Metabolic syndrome: case (metabolic syndrome and pre-metabolic syndrome), non-case (non-metabolic syndrome)

^c^*P*-value of the metabolic syndrome in extended Fisher's exact test (all over test). Boldface indicates statistically significant differences

^d^Fisher's exact test for multiple comparisons: *P* < 0.001 between I-type and D-type

### Risk factors for metabolic syndrome according to with/without physical activity

The risk factors for metabolic syndrome with/without physical activity differed by sex (Table 4). Women with exercise showed significantly lower LDL-C levels (*P* = 0.001), whereas those with daily physical activity had significantly lower values of the five risk factors: BMI, waist circumference, systolic blood pressure, LDL-C, and HbA1c. There were no significant differences among the male participants (Table 4).

**Table 4:** Mean values of risk factors for metabolic syndrome in analyses stratified by sex and physical activity

	Men (*n* = 269)					Women (*n* = 367)				
	With		Without		*P*^*b*^	With		Without		*P*^*b*^
	Mean	(SD)	Mean	(SD)		Mean	(SD)	Mean	(SD)	
**Q1. Exercise**^**a**^**,** ***n***	104		165			121		246		
Body mass index, kg/m^2^	23.3	(3.0)	23.3	(3.0)	0.809	21.9	(3.2)	22.1	(3.6)	0.600
Waist circumference, cm	84.7	(7.8)	85.1	(8.2)	0.741	81.6	(9.6)	81.3	(10.0)	0.932
Systolic blood pressure, mmHg	133.0	(15.7)	130.3	(17.0)	0.279	129.7	(15.0)	129.4	(16.5)	0.463
Diastolic blood pressure, mmHg	76.6	(10.4)	76.4	(11.6)	0.742	73.0	(10.4)	73.4	(10.5)	0.509
Triglyceride, mg/dL	135.8	(100.7)	135.3	(79.6)	0.898	99.8	(55.0)	103.7	(58.4)	0.430
HDL-C, mg/dL	59.3	(13.9)	57.6	(16.8)	0.357	67.8	(15.9)	69.2	(16.7)	0.482
LDL-C, mg/dL	113.8	(30.5)	120.0	(30.7)	0.261	112.4	(25.5)	122.8	(31.1)	**0.001**
HbA1c, %	5.9	(0.7)	6.0	(0.9)	0.226	5.8	(0.4)	5.8	(0.5)	0.222
**Q2. Daily physical activity**^**a**^**,** ***n***	142		127			173		194		
Body mass index, kg/m^2^	23.1	(3.0)	23.6	(3.3)	0.248	21.4	(3.2)	22.6	(3.6)	**0.001**
Waist circumference, cm	84.2	(7.2)	85.9	(8.9)	0.102	79.6	(9.2)	82.9	(10.1)	**< 0.001**
Systolic blood pressure, mmHg	131.1	(15.8)	131.6	(17.3)	0.629	127.9	(16.9)	131.0	(15.1)	**0.039**
Diastolic blood pressure, mmHg	75.9	(11.4)	77.2	(10.8)	0.402	72.4	(10.2)	74.1	(10.7)	0.104
Triglyceride, mg/dL	126.4	(81.6)	145.6	(94.4)	0.086	96.6	(55.2)	107.6	(58.6)	0.064
HDL-C, mg/dL	59.9	(14.2)	56.4	(17.2)	0.070	70.2	(15.8)	67.4	(16.8)	0.097
LDL cholesterol, mg/dL	118.5	(29.7)	116.6	(31.9)	0.331	113.5	(27.2)	124.7	(30.9)	**< 0.001**
HbA1c, %^b^	5.9	(0.7)	6.1	(1.0)	0.268	5.7	(0.4)	5.8	(0.5)	**0.014**

*HDL-C* high-density lipoprotein cholesterol, *LDL-C* low-density lipoprotein cholesterol, *HbA1c* glycated hemoglobin

^a^“Q1. Exercise” and “Q2. Daily physical activity”: see Table 1

^b^*P* independent *t*-test adjusted by age. Boldface indicates statistically significant differences

### Association between risk factors for metabolic syndrome according to physical activity type

Table 5 shows the mean differences in the prevalence of risk factors for metabolic syndrome according to the physical activity type. F-type women had significantly lower LDL-C levels than I-type women. Furthermore, participants in the D-type group had significantly lower BMI, lower waist circumference, and higher HDL-C measurements than those in the I-type group.

**Table 5:** Mean differences in the risk factors for metabolic syndrome by physical activity type in sex-stratified analyses

		Without daily physical activity				With daily physical activity				
		Without exercise		With exercise		Without exercise		With exercise		
		(I-type)^**a**^		(E-type)^**a**^		(D-type)^**a**^		(F-type)^**a**^		
	***n***	mean	SD	mean	SD	mean	SD	mean	SD	***P***^***b***^
**Men (*n*** **= 269)**		I-type (*n* = 112)		E-type (*n* = 15)		D-type (*n* = 53)		F-type (*n* = 89)		
Body mass index, kg/m^2^	269	23.5	3.2	24.4	4.0	23.0	2.7	23.1	2.8	0.354
Waist circumference, cm	269	85.7	8.7	86.8	10.2	83.9	6.8	84.4	7.4	0.334
Systolic blood pressure, mmHg	269	131.1	17.3	135.5	17.9	128.5	16.4	132.6	15.3	0.472
Diastolic blood pressure, mmHg	269	77.0	10.9	78.7	10.7	75.2	13.0	76.3	10.3	0.669
Triglyceride, mg/dL	269	142.9	86.4	143.1	68.0	119.4	60.7	130.7	91.8	0.203
HDL-C, mg/dL	269	56.7	17.4	54.1	16.0	59.3	15.5	60.2	13.4	0.291
LDL-C, mg/dL	269	118.1	31.3	105.3	35.1	124.0	29.2	115.3	29.6	0.162
HbA1c, %	234	6.1	1.0	5.8	0.5	5.9	0.6	5.9	0.7	0.431
**Women (*n*** **= 367)**		I-type (*n* = 162)		E-type (*n* = 32)		D-type (*n* = 84)		F-type (*n* = 89)		
Body mass index, kg/m^2^	367	22.6^c^	3.8	22.6	2.8	21.2^c^	3.0	21.7	3.4	**0.008**
Waist circumference, cm	367	82.8^d^	10.5	83.4	8.3	78.2^d^	8.1	81.0	10.0	**0.003**
Systolic blood pressure, mmHg	367	130.7	15.1	132.1	15.4	126.9	18.8	128.8	14.9	0.243
Diastolic blood pressure, mmHg	367	73.9	10.2	74.9	10.2	72.4	11.0	72.3	10.4	0.443
Triglyceride, mg/dL	367	108.0	58.8	105.3	58.3	95.4	56.9	97.8	53.9	0.328
HDL-C, mg/dL	367	67.1^e^	16.1	68.7	20.5	73.2^e^	17.2	67.4	14.0	**0.043**
LDL-C, mg/dL	367	125.4^f^	30.8	121.0	31.7	117.8	31.2	109.3^f^	22.2	**0.002**
HbA1c, %	302	5.8	0.5	5.9	0.6	5.7	0.4	5.7	0.3	0.116

*HDL-C* high-density lipoprotein cholesterol, *LDL-C* low-density lipoprotein cholesterol, *HbA1c* glycated hemoglobin

^a^I-type, D-type, E-type, and F-type: see Table 1

^b^*P* One-way analysis of variance adjusted by age. Boldface indicates statistically significant differences

Tukey’s honest significant difference test for multiple comparisons: ^c^*P* < 0.01 between I-type and D-type; ^d^*P* < 0.01 between I-type and D-type; ^e^*P* < 0.05 between I-type and D-type; ^f^*P* < 0.001 between I-type and F-type

### ORs of physical activity types to prevent metabolic syndrome by sex-specific measures

As shown in Table 6, logistic regression analysis revealed that women in the D-type group were less likely to have metabolic syndrome (AOR = 0.24, 95% CI, 0.06–0.85) than those in the E-type group. However, a significant result was not observed for men.

**Table 6:** Sex-stratified odds ratios of physical activity types for the prevention of metabolic syndrome

	Metabolic syndrome^a^															
	Men (*n* = 68)								Women (*n* = 116)							
	OR	95% CI		*P*	AOR	95% CI		*P*	OR	95% CI		*P*	AOR	95% CI		*P*
E-type^b^	1.00	(reference)			1.00	(reference)			1.00	(reference)			1.00	(reference)		
D-type^b^	0.65	0.30	1.46	0.285	1.21	0.37	4.37	0.753	0.23	0.30	0.77	**0.018**	0.24	0.06	0.85	**0.028**

Boldface indicates statistically significant differences

*OR* odds ratio, *AOR* adjusted odds ratio by age, *CI* confidence interval

^a^The dependent variables are defined by “the presence of metabolic syndrome or pre-metabolic syndrome: case” and “non-metabolic syndrome: non-case”

^b^The independent variable is the type of physical activity. D-type and E-type: see Table 1

## Discussion

This study examined whether daily physical activity without exercise had a higher association with the prevention of metabolic syndrome than exercise without daily physical activity in middle-aged and older participants in a semi-mountainous area of Japan. Overall, the prevalence of metabolic syndrome in this study had a large sex-specific difference similar to national prevalence, but both sex prevalence in this study was higher than the national prevalence level: 45.7%, 95% CI 42.7 to 48.7 in men and 15.8%, 95% CI 13.9 to 17.7 in women. For the national average, the prevalence of men and women was 39.9% and 11.8%, respectively in 2017 [27]. This is because our study had a higher proportion of older participants than the national average. In this study, the proportion of participants over 60 years old was 86.6%, while that of total examinees in Japan was 35.9% [27]. Furthermore, regarding Japanese employees’ medical insurance, the specific health checkup examinations of those over 60 years was 19.8%, and the total prevalence of metabolic and pre-metabolic syndrome was 38.3% and 9.8%, in men and women, respectively [27]. The older adults are more susceptible to metabolic syndrome due to decreased glucose tolerance with age [28] and decreased basal metabolism [29].

The main finding of this study was that middle-aged and older women with D-type activity (with daily physical activity and without exercise) were less likely to have metabolic syndrome than those who had E-type activity (without daily physical activity and with exercise) (Table 6). In contrast, the difference was not significant among men. This result could potentially be attributed to the following three possible reasons:

First, exercise does not affect the total amount of physical activity of middle-aged and elderly individuals. Since they might have been exhausted after exercise and unable to remain active, the resulted total amount of physical activity per day did not change. This phenomenon was found elsewhere [30–32]. Exercise had a limited association with metabolic syndrome and its risk factors, that is, exercise affected only one risk factor (Table 4) and did not affect metabolic syndrome (Table 3). Thus, to prevent metabolic syndrome through increased physical activity in middle-aged and older women, we recommend promoting daily physical activity rather than an increase in exercise. A previous study has shown that active daily life was important to prevent obesity, independent of exercise [33]. Similarly, active daily life might be important to prevent metabolic syndrome in women. This suggestion is consistent with the recommendation by the World Health Organization [13] and the Japanese MHLW [7], which encourages people to be more active in daily life for a long and healthy life.

Second, the characteristics of the daily physical activity of women may differ from those of the physical activity of men. The question used in our study, Q2 “Do you walk for at least 1 h every day or have equivalent physical activities in your daily life?” includes activities that are undertaken daily, e.g., housework [23]. The intensity of physical activity of housework ranges from light to moderate–vigorous [34]. Women spend longer durations in housework than men [35–37]. More than that of men, the lifestyle of women ensures that they undertake more frequent light to moderate–vigorous physical activities [38]. A study has shown that housework enhances the association of physical activity with metabolic syndrome by increasing the total energy expenditure [39]. Other studies have found that light intensity [40, 41] or more than 10 min of consecutive moderate–vigorous physical activity [42] prevents metabolic syndrome.

Third, unlike flat urban or suburban areas, a semi-mountainous area has many slopes and steps, enhancing routine up and down movement on foot or by bicycle, leading to a higher intensity of daily physical activity [34]. Furthermore, the presence and availability of destinations, such as commercial and leisure facilities in the neighborhood, are associated with physical activity promotion in older adults [43, 44]. However, few destinations are available in semi-mountainous areas [45]; the only destinations available may be their own farms or the houses of their neighbors. In the daily life of female residents of semi-mountainous areas, active daily agricultural farm work for daily meals, gardening a larger yard, and visiting distant neighbors may enhance daily physical activity.

In our study, it is possible that daily physical activity increased the total energy expenditure of women involved in housework or that light-intensity daily physical activity and moderate–vigorous physical activity derived from housework were related to the prevention of metabolic syndrome. Thus, an intervention focusing on household chores is a promising way to prevent metabolic syndrome among women in semi-mountainous areas.

This study suggests that enhancing daily physical activity may be a key to preventing metabolic syndrome in middle-aged and older individuals residing in semi-mountainous areas of Japan. A more detailed evaluation of the daily physical activities of women, including content (e.g., housework), intensity, and total time of daily physical activity, is necessary in further research.

This study has several potential limitations. First, we could not explain the causal relationship between the daily physical activity and metabolic syndrome and its risk factors because of the cross-sectional design. Second, this study used data from a self-reported questionnaire, which could not assess the actual accurate amount and intensity, namely metabolic equivalents (METs), total time, and content of physical activities. Although the validity of the questionnaire has been confirmed and is used officially in Japan, the accuracy is rather limited [46]. Third, the generalizability of the study findings could be limited because our study examined only one area. In further research, emphasis should be placed on objective physical activity measurements, including light-intensity physical activity, to assess the association between daily physical activity and metabolic syndrome and its risk factors.

## Conclusions

Among those living in a semi-mountainous area of Japan, middle-aged and older women with daily physical activity and without exercise had lower BMIs, smaller waist circumferences, higher HDL-C levels, and a lower prevalence of metabolic syndrome than those without daily physical activity and with exercise. Compared to middle-aged and older women with exercise in this region, those with daily physical activity may effectively prevent metabolic syndrome.

## Data Availability

The datasets analyzed during the current study are not publicly available due to the necessity of permission from the town. They are not allowed for secondary usage.

## Abbreviations

AOR: Adjusted odds ratio
BMI: Body mass index
CI: Confidence interval
HbA1c: Glycated hemoglobin
HDL-C: High-density lipoprotein cholesterol
LDL-C: Low-density lipoprotein cholesterol
MHLW: Ministry of Health, Labour and Welfare
OR: Odds ratio
SD: Standard deviation
